# Effects of Jasmonate on Ethylene Function during the Development of Tomato Stamens

**DOI:** 10.3390/plants8080277

**Published:** 2019-08-09

**Authors:** Ramona Schubert, Stephan Grunewald, Lea von Sivers, Bettina Hause

**Affiliations:** Department of Cell and Metabolic Biology, Leibniz Institute of Plant Biochemistry, D06120 Halle (Saale), Germany; Ramona.Schubert@ipb-halle.de (R.S.), Stephan.Grunewald@ipb-halle.de (S.G.), Lea_von_Sivers@gmx.de (L.v.S)

**Keywords:** ethylene, gene expression, jasmonate, nanofluidic arrays, phenylpropanoids, pollen vitality, *Solanum lycopersicum* (tomato), stamen development

## Abstract

The phenotype of the tomato mutant *jasmonate-insensitive1-1* (*jai1-1*) mutated in the JA-Ile co-receptor COI1 demonstrates JA function in flower development, since it is female-sterile. In addition, *jai1-1* exhibits a premature anther dehydration and pollen release, being in contrast to a delayed anther dehiscence in the JA-insensitive Arabidopsis mutant *coi1-1*. The double mutant *jai1-1 Never ripe* (*jai1-1 Nr*), which is in addition insensitive to ethylene (ET), showed a rescue of the *jai1-1* phenotype regarding pollen release. This suggests that JA inhibits a premature rise in ET to prevent premature stamen desiccation. To elucidate the interplay of JA and ET in more detail, stamen development in *jai1-1 Nr* was compared to wild type, *jai1-1* and *Nr* regarding water content, pollen vitality, hormone levels, and accumulation of phenylpropanoids and transcripts encoding known JA- and ET-regulated genes. For the latter, RT-qPCR based on nanofluidic arrays was employed. The data showed that additional prominent phenotypic features of *jai1-1*, such as diminished water content and pollen vitality, and accumulation of phenylpropanoids were at least partially rescued by the ET-insensitivity. Hormone levels and accumulation of transcripts were not affected. The data revealed that strictly JA-regulated processes cannot be rescued by ET-insensitivity, thereby emphasizing a rather minor role of ET in JA-regulated stamen development.

## 1. Introduction

Flower development is controlled by several plant hormones including their cross talk [1]. Among these hormones involved in regulation of flower development are jasmonic acid (JA) and its derivatives, commonly named jasmonates [2]. Jasmonates are lipid-derived compounds that ubiquitously occur in higher plants and also act in the plant’s response to biotic and abiotic stress [2,3]. JA biosynthesis starts from α-linolenic acid, which is released from plastid membranes, via the ALLENE OXIDE SYNTHASE (AOS) branch of the LIPOXIGENASE (LOX) pathway [4]. The main intermediate produced in plastids, *cis*-12-oxophytodienoc acid (OPDA), is converted within peroxisomes to JA, which is further metabolized into (+)-7-*iso*-jasmonoyl isoleucine (JA-Ile) in the cytosol [5]. JA-Ile represents the most biologically active form of jasmonates in higher plants [6]. JA-Ile mediates interaction of the co-receptor proteins CORONATINE INSENSITIVE1 (COI1) and JASMONATE ZIM DOMAIN (JAZ) [6,7,8,9], thereby mediating the proteasomal degradation of JAZ proteins and freeing transcription factors, such as MYC2, from repression to enable transcription of JA-induced genes [2,3,10].

Mutant plants defective in the co-receptor protein COI1 are insensitive to JA-Ile and show severe defects in flower development leading to sterility. The *Arabidopsis thaliana* mutant *coi1* is male sterile and does not produce seeds due to insufficient elongation of stamen filaments and dehiscence of anthers as well as to non-germinating pollen [11,12]. The orthologous mutant in *Solanum lycopersicum* (tomato) called *jasmonate-insensitive1-1* (*jai1-1*) shows defects in the development of ovules and is therefore female-sterile [13,14,15]. In addition, *jai1-1* plants also exhibit defects in male reproductive function, such as a reduction in pollen viability and germination. This does not result, however, in male sterility of *jai1-1* flowers, since the pollen is still capable to fertilize wild-type flowers [14]. The flower development in *jai1-1* is very similar to the wild type. All stages ranging from small buds completely enclosed by sepals up to the mature/biggest bud-stage showing slightly opened sepals and greenish-white petals appear to be very similar in both genotypes [16]. In contrast, the open flowers differ between wild type and *jai1-1* by prominent phenotypic features of *jai1-1*, such as the protrusion of the stigma out of the stamen cone and the senescing tip of the stamen cone [14,16].

Comparison of *jai1-1* and WT stamens in terms of gene expression and metabolite accumulation revealed that jasmonates are involved in the regulation of pollen nutrition and pollen development at early stages of stamen development [16]. At later stages, however, jasmonates appear to be an important regulator of the proper timing of stamen dehiscence, since the senescence phenotype of *jai1-1* stamens is accompanied by premature dehiscence and pollen release [16]. The process of dehiscence occurs in specialized cells of the anther that determine the site of anther opening/pollen release (for review see [17]). Localized degeneration of the stomium and dehydration [18] is regulated by the plant hormone ethylene (ET) [17,19]. In *jai1-1*, genes encoding enzymes involved in ET biosynthesis, ET-related TFs as well as ET-response genes are expressed earlier during stamen development than in wild type. This pre-mature ET function might cause the enhanced dehiscence of the *jai1-1* stamen in the not-yet opened flower and mis-regulated pollen release [16]. First cross-talk analyses between JA and ET have supported this hypothesis: On the one hand, JA-mediated effects on ET biosynthesis and function were analyzed in JA-deficient (*SlAOC*-RNAi) plants. Here, treatment of flower buds with JA resulted in decreased transcript levels of genes encoding ET biosynthetic enzymes [16]. On the other hand, the ethylene insensitive mutant *Never ripe* (*Nr*) lacking petal and stamen senescence was used for the investigation [20]. This mutant carries a non-synonymic point mutation in ETR3 encoding gene (*Solyc09g075440*), leading to an exchange of proline to leucine [21]. The *Nr* mutation is partially dominant and leads to a loss of the capacity to respond to either endogenously generated or exogenously applied ET in all plant tissues [20]. Crosses between *jai1-1* and *Nr*, leading to JA- and ET-insensitive double mutant plants showed a complementation of the *jai1-1* phenotype in terms of pollen release [16].

These data suggested an essential role of jasmonates in the temporal inhibition of ET production to prevent premature desiccation of stamens and to ensure proper timing in flower development. This contrasts to Arabidopsis, where both hormones act in parallel to regulate timing of floral organ abscission [22]. Also in plant’s responses to stress, jasmonates and ET might act cooperatively, such as during plant’s response to necrotrophic pathogens [23]. However, there are also antagonistic actions of both hormones described, such as their role in the regulation of the expression of wound-responsive and metabolite biosynthetic genes [24,25].

To get insights into the cross-talk between JA and ET during stamen development of tomato, we compared developing stamen of wild type, *jai1-1*, *Nr* and *jai1-1 Nr* regarding phenotypic features, such as pollen vitality and water content of stamen, accumulation of secondary metabolites and gene expression of selected JA- and ET-related genes. Although phenotypic alterations of *jai1-1* were largely rescued by the insensitivity to ET, the double mutant *jai1-1 Nr* was more similar to *jai1-1* in terms of gene expression and metabolite accumulation, thereby differing from wild type and *Nr*. 

## 2. Results and Discussion

### 2.1. Diminished Water Content of jai1-1 Is Restored by Ethylene Insensitivity

**Figure 1:**
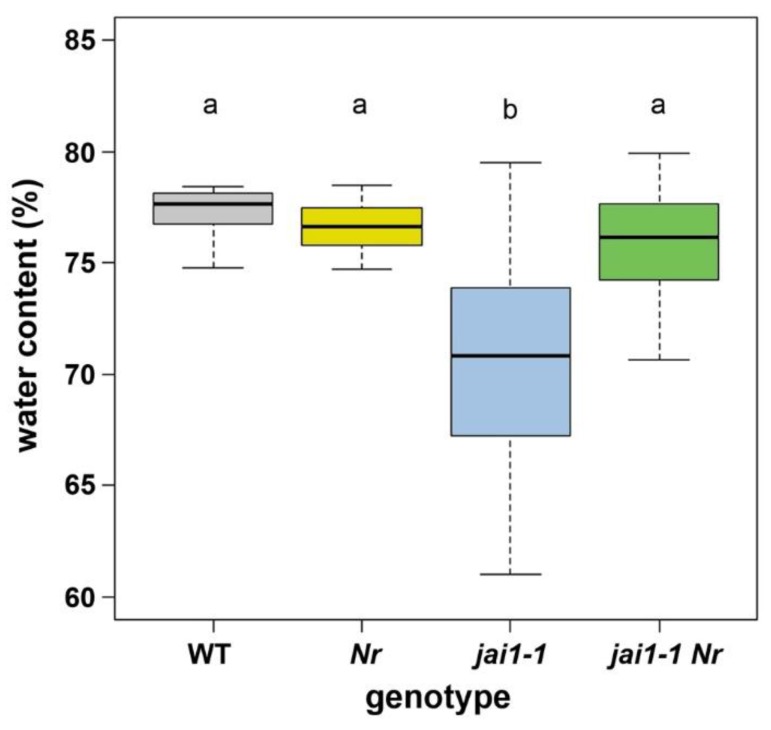
Water content of stamens from wild type (WT), *Nr*, *jai1-1* and *jai1-1 Nr*. Water content was determined from stamens dissected from flowers at developmental stage 6 (open flower) with n = 20–28. Boxplots show median (crossbar), 25–75 % interquartile range (boxes) and data distribution (error bars). Different letters designate statistically different values (1-factorial ANOVA followed by Tukey’s HSD test; *p* ≤ 0.05).

Open flowers (stage 6) from *jai1-1* plants show a desiccated tip of the anther cone accompanied by a significantly reduced water content in comparison to stamens of wild-type plants [16]. To elucidate, whether the postulated pre-mature biosynthesis and action of ethylene (ET) is responsible for this phenotypic feature, *jai1-1* was crossed with *Nr*, an ET-insensitive mutant, to obtain the double mutant *jai1-1 Nr*, which is JA- and ET-insensitive [16]. Determination of water content from stamens of all genotypes revealed that stamens from wild type and *Nr* exhibited a water content in the range of 76–78%, whereas stamens of *jai1-1* showed a water content of about 70% (Figure 1). Most importantly, water content of stamens from *jai1-1 Nr* double mutant plants was not significantly different to stamens from wild type and *Nr*, thereby significantly different from *jai1-1*. This result shows that a loss of ET function accomplished by the ET insensitivity in *jai1-1 Nr* led to a rescue of the premature water loss visible in stamens of *jai1-1*. Moreover, it supports the hypothesis that the phenotypic alterations in late stages of stamen development caused by JA insensitivity in *jai1-1* are due to a pre-mature function of ET. 

A role of ET in senescence processes during flower development was demonstrated since ET insensitivity increases flower lifetime in several ornamental species [26]. More specifically, it is involved in timing of the anther dehiscence in *Solanaceae* species, such as tobacco and petunia [19,27]. Surprisingly, the *Nr* mutant is not impaired in water content of the stamens (Figure 1) and timing of pollen release [16]. These data suggest that in tomato, ET is mainly regulating processes occurring shortly before and after anthesis. Nevertheless, any pre-mature function of ET is repressed by jasmonates. This is in strong contrast to Arabidopsis, where jasmonates serve as important regulators to trigger anther dehiscence visible in the delay of this process in the *coi1* mutant [11]. 

### 2.2. Diminished Pollen Vitality of Jai1-1 Is Partially Restored by Ethylene Insensitivity

**Figure 2:**
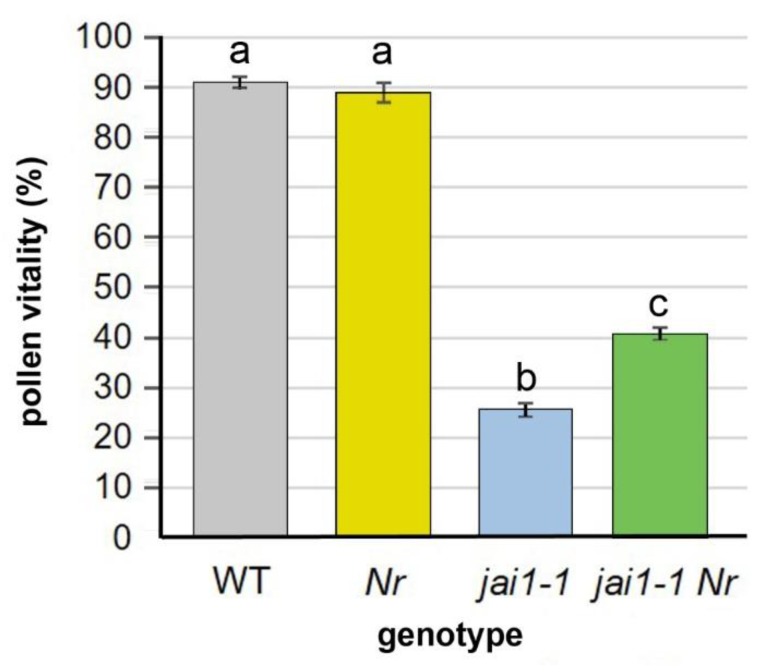
Pollen vitality in open flowers from wild type (WT), *Nr*, *jai1-1* and *jai1-1 Nr*. Pollen from flowers at developmental stage 6 (open flower) were isolated and stained with fluorescein-diacetate. Vitality was calculated from the ratio of living and dead pollen. At least 900 pollen were analyzed per biological replicate. Data are given as mean values ± SE (n = 5). Different letters designate statistically different values (1-factorial ANOVA followed by Tukey’s HSD test; *p* ≤ 0.05). Representative micrographs are shown in Figure S1.

In addition to the premature dehiscence and pollen release, *jai1-1* plants are characterized by production of a reduced number of mature, living pollen compared to wild type [14,16]. To test whether the cross-talk between JA and ET affects pollen vitality, the rate of vital pollen from open flowers of *jai1-1*, *Nr* and *jai1-1 Nr* was determined in comparison to wild type (Figure 2). As already known, the vitality of pollen from *jai1-1* was highly reduced in comparison to wild type [14,16], whereas stamens of *Nr* exhibited a similar ratio of vital pollen as the wild type. The double mutant *jai1-1 Nr* contained a significantly reduced number of vital pollen in comparison to wild type, but a significantly higher number than *jai1-1* (Figure 2). This showed that ET insensitivity results in an at least partial rescue of the pollen vitality. 

A cross-talk between JA and ET in early pollen development was excluded due to the facts that JA insensitivity affects early stage of stamen and pollen development by defective nutrition of microspores and that the rise of ET was detectable at later developmental stages characterized by declining JA levels [16]. Indeed, the effect of JA insensitivity on pollen vitality seems to be dominant, since the pollen vitality in *jai1-1 Nr* is much lower than in wild type (Figure 2). This might be due to premature degeneration of tapetum on the one hand, and to the absence of fluids in the locule of *jai1-1* stamen on the other hand [16]. However, *jai1-1 Nr* flowers exhibited in an improved pollen vitality in comparison to *jai1-1*. It is tempting to speculate that rescued timing of dehiscence supports to some extent pollen nutrition by providing nutritive saps in the locule. Although a functional tapetum is most critical for proper microspore and pollen development [28], developing pollen grains are immersed in the locular fluid, which also supports pollen nutrition [29].

### 2.3. Hormone Levels in Developing Stamen 

**Figure 3:**
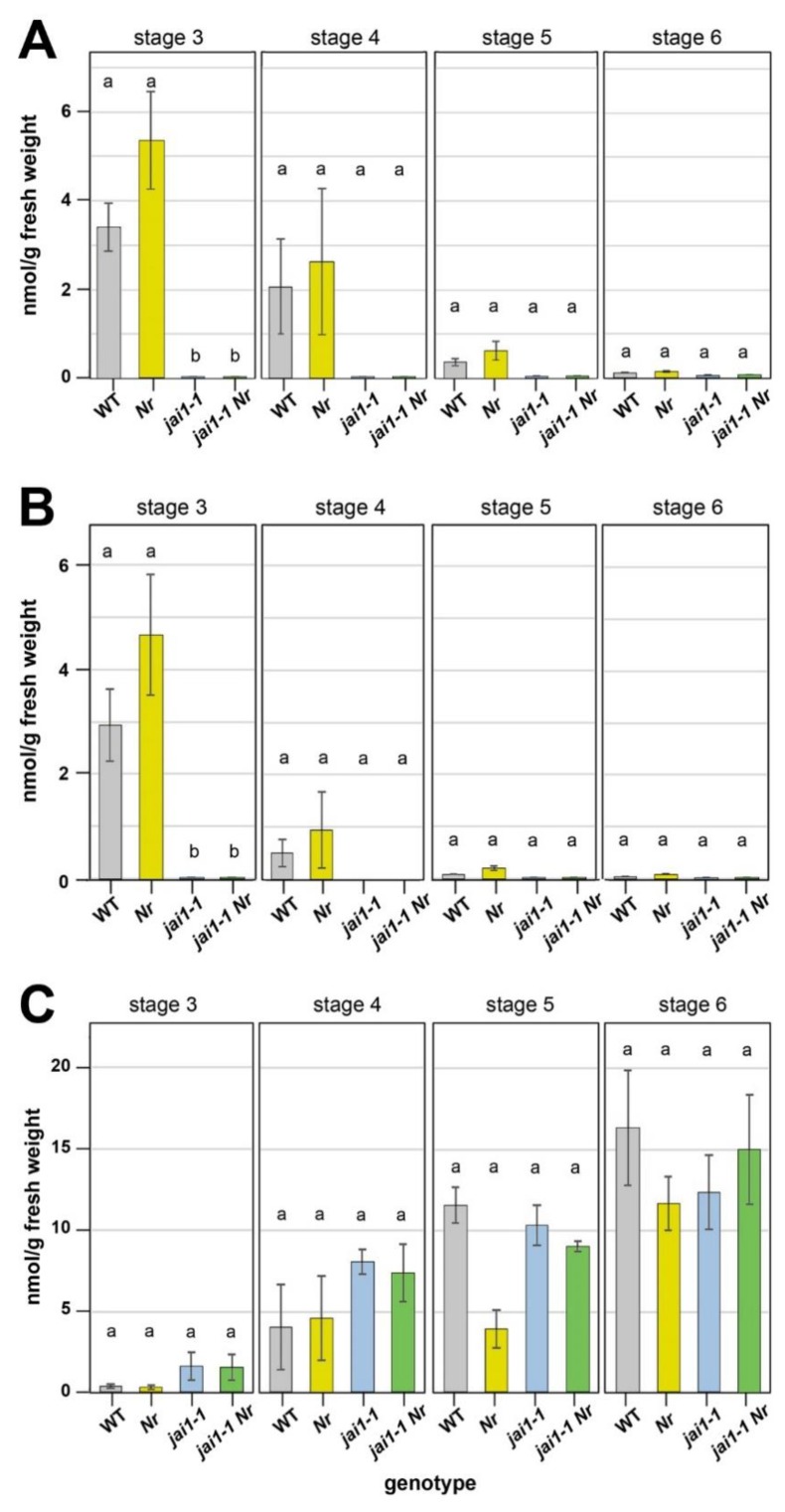
Hormone levels in developing stamens of wild type (WT), *Nr*, *jai1-1* and *jai1-1 Nr*. Stamens of the respective stages were dissected and contents of JA (**A**), JA-Ile (**B**) and ACC (**C**) were determined. Data are given as mean values ± SE (n = 4). Data from the same developmental stage were compared by 1-factorial ANOVA, followed by Tukey’s HSD test and different letters designate statistically different values (*p* ≤ 0.05).

The *Nr* mutant is ET insensitive due to a block in ET perception, but ET biosynthesis is not affected in these plants [20]. To test if the double mutant *jai1-1 Nr* behaves accordingly, levels of 1-aminocyclopropane-1-carboxylic acid (ACC), JA and JA-Ile were measured in stamens of the flower stages 3 to 6. These developmental stages were characterized previously from wild type and *jai1-1*, whereby stage 3 stamens of wild type showed the maximum in accumulation of JA and JA-Ile [16]. Similar to these data, highest levels of JA and JA-Ile were detected in stage 3 of wild-type stamens, but at the same levels also in *Nr* (Figure 3A,B). In stamens from *jai1-1* and *jai1-1 Nr* plants, almost no JA and JA-Ile were detectable throughout the complete development from stage 3 to 6. This might be due to the missing expression of genes encoding JA biosynthesis genes, which are known to be regulated by JA and thereby contributing to the positive feedback in JA biosynthesis [2]. 

The levels of the ET precursor ACC increased in all genotypes during the development from flower stage 3 to 6 (Figure 3C). There were no significant differences detectable between the four genotypes within each stage, but by trend higher ACC-levels were measured in stamens from *jai1-1* and *jai1-1 Nr* at stages 3 and 4. From these data, it is to conclude that the double mutant *jai1-1 Nr* is highly similar to the *jai1-1* mutant in terms of JA, JA-Ile and ACC levels. Therefore, the observed rescue in water content seems to be caused rather by hormone signalling than by altered hormone levels. This is consistent with the fact that—depending on physiological responses—reduced JA levels might cause ET sensitivity in originally ET insensitive mutants [30]. Here, JA affects ET signalling downstream of the ET receptors [30]. Moreover, it has been shown for apical hook formation on Arabidopsis that MYC2, a prominent transcription factor activated by JA, can physically interact with the ET-activated transcription factor ETHYLENE INSENSITIVE3 to antagonize ET function [31,32]. It is tempting to speculate that the missing activity of MYC2 in stamen of *jai1-1* contributes to the activation of the ET-signalling pathway, which is abolished in the double mutant *jai1-1 Nr*. 

### 2.4. Accumulation of Phenylpropanoids

**Figure 4:**
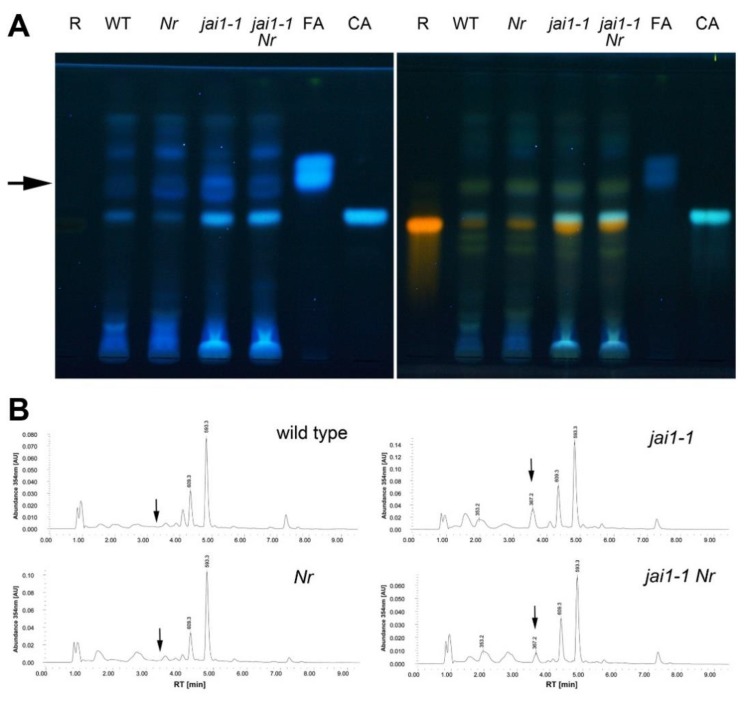
Accumulation of phenylpropanoids in stamens of open flowers from wild type (WT), *Nr*, *jai1-1* and *jai1-1 Nr* as analyzed by high-performance thin-layer chromatography (HPTLC) and liquid chromatography-mass spectroscopy (LC-MS). A. Methanolic extracts from stamens dissected from flowers at developmental stage 6 (open flower) were separated by HPTLC and derivatized using diphenylboric acid 2-aminoethylester (DPBA). Pictures were taken under illumination at 366 nm before (left) and after (right) derivatization with DPBA. Rutin (R), feruloylquinic acid (isomeric mix, FA) and chlorogenic acid (CA) served as authentic standards. B. Chromatograms obtained by LC-MS show the main phenylpropanoids accumulating in stamens of all four genotypes. The *m*/*z* ratios of chlorogenic acid (353.2 *m*/*z*), feruloylquinic acid (367.2 *m*/*z*), rutin (609.3 *m*/*z*) and a kaempferol rutinoside (593.3 *m*/*z*) are given (for chromatograms of the authenic standards rutin and feruloylquinic acid see Figure S2). Rutin (609.3 *m*/*z*) accumulates in stamens of all genotypes, whereas feruloylquinic acid (367.2 *m*/*z*) is not detectable in wild type and *Nr* stamen, but in stamen of *jai1-1* and *jai1-1 Nr* (arrows in **A** and **B**). Note that the levels of feruloylquinic acid in stamens of the double mutant *jai1-1 Nr* are lower in comparison to stamens of *jai1-1*.

Jasmonates are involved in the regulation of the biosynthesis of phenylpropanoids in flowers of tomato, because treatments of leaves with JA elicits massive accumulation of caffeoylputrescine in leaf tissue, but have only little or no effect on the levels of rutin and chlorogenic acid [33]. Also flowers from tomato wild-type plants contain relatively high levels of caffeoylputrescine, which were not detectable in *jai1-1* flowers. To characterize stamens in more detail, accumulation of phenolic compounds was analyzed in stage 6 of wildtype, *Nr*, *jai1-1* and *jai1-1 Nr* using high performance thin-layer chromatography (HPTLC) and liquid chromatography-mass spectroscopy (LC-MS). The results revealed a similar occurrence of detected phenylpropanoids between all genotypes (Figure 4A). 

There were only few quantitative differences between stamen extracts from wild type and *jai1-1*, the latter showing higher levels of rutin, feruloylquinic acid and chlorogenic acid. The phenylpropanoid pattern of stamens from *Nr* was highly similar to wild type, whereas the pattern from the double mutant was more similar to *jai1-1*. There was, however, one exception: Levels of feruloylquinic acid, which were detectable in *jai1-1* but only in traces in wild type and *Nr*, showed an intermediate level in *jai1-1 Nr* pointing to an at least partial rescue of *jai1-1* by ET insensitivity regarding this compound (arrows in Figure 4B). Here, the abundance at 354 nm was reduced from 0.035 arbitrary units (AU) in *jai1-1* to 0.008 AU in *jai1-1 Nr*.

Changes in the phenylpropanoid content in stamens from *jai1-1* and *jai1-1 Nr* in comparison to stamens from wild type and *Nr* might contribute to defects in stamen development in the JA-insensitive mutants. Phenylpropanoids and especially flavonoids are involved in pollen development in many species of the Solanaceae family [34,35]. In contrast to caffeoyl putrescine, which is strictly JA-dependent when produced in tomato flowers, chlorogenic acid and rutin were found to accumulate to higher levels in stamen from *jai1-1* flowers (Figure 4). The enhanced accumulation of chlorogenic acid and rutin in stamen from *jai1-1* and *jai1-1 Nr* might occur exclusively in stamen and might therefore not be visible in extracts from complete flowers [33]. One product of chlorogenic acid metabolism is feruloylquinic acid. This compound was almost undetectable in wild-type and *Nr* stamen, but occurred in stamen from *jai1-1*. In comparison to this, its amount was decreased in stamens from the double mutant *jai1-1 Nr* correlating with the observed (partial) rescue in water content and pollen vitality. On the one hand, chlorogenic acid is involved in the regulation of the expression of *phenylalanine ammonia-lyase* (*PAL*) encoding the enzyme, which catalyzes the first step in the phenylpropanoid synthesis, thereby redirecting flux of phenylpropanoids [36]. On the other hand, phenolic compounds can be conjugated to hydroxy fatty acids leading to the formation of suberin [37], which then might function in the anther cuticle to protect anthers against water loss [38]. It is tempting to speculate that the accumulation of phenolic compounds in *jai1-1* is due to missing conjugation to hydroxy fatty acids and might be therefore closely related to or even cause defects in dehiscence.

### 2.5. Transcript Accumulation of JA and ET Related Genes

The results shown above point to a rescue of *jai1-1* phenotype by ET insensitivity mainly in late stamen development. Therefore, high throughput qPCR analysis was conducted for wildtype, *Nr*, *jai1-1* and *jai1-1 Nr* stamens from flower stage 3 to 6 addressing genes, which are regulated by JA during late stamen development [16]. Genes of interest were selected from the previously published comparative transcriptomics data of wild type and *jai1-1* stamens regarding differentially regulated transcription factors, JA- and ET-regulated genes as well genes involved in protein degradation and carbohydrate metabolism [16]. In total, 47 genes were selected, but only 24 amplified a specific PCR-product using Fluidigm HD technology. We have chosen this relatively new and flexible method, because it has a medium to high throughput and needs amounts of cDNA that are 70–150 times smaller than those used for conventional RT-qPCR [39]. In our hands, however, the number of data points was relatively high that did not fulfil the quality criteria of the system.

**Figure 5:**
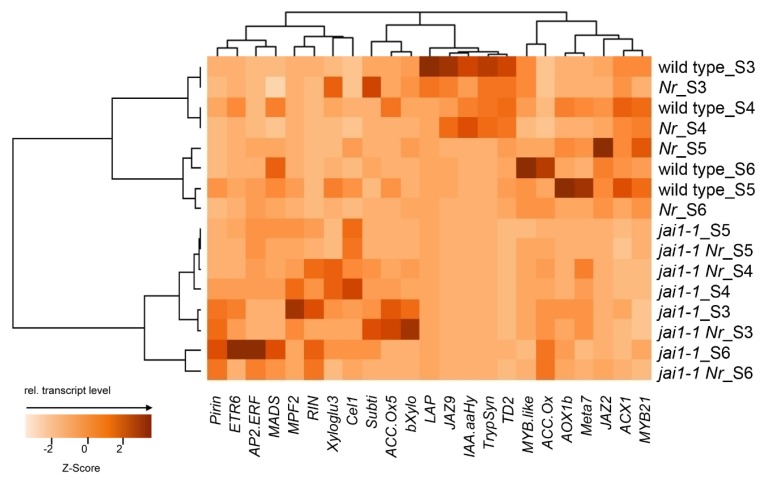
Comparative analysis of transcript accumulation in developing stamens of wild type, *Nr*, *jai1-1* and *jai1-1 Nr*. The heatmap with column-scaled normalization of the means of the relative expression values of selected genes obtained by the BioMark™ system with a “48.48 Dynamic Array Chip for Gene Expression” is shown. The analyzed genes are given at the x-axis, whereas the genotype and developmental stage (abbreviated as S3, S4, S5 and S6) are presented at the y-axis. As distance measure for the hierarchical clustering of genotypes and stages served the Pearson correlation coefficient with complete linkage. The calculated, relative transcript levels are given in Table S1.

Hierarchical cluster analysis using the means of the relative expression values was performed to visualize the similarity in gene expression between the four genotypes (Figure 5). It revealed two distant clusters containing wild type and *Nr* in one and *jai1-1* and *jai1-1 Nr* in the other branch, thereby showing that the double mutant is more similar to *jai1-1* than to *Nr* or wild type. Half of the 24 genes showed significant differences in transcript accumulation within the four genotypes in at least one developmental stage (Table S1). Most of these genes showed a similar expression pattern in wild type and *Nr*, and some of them were almost not expressed in stamens of *jai1-1* and *jai1-1Nr* (Table S1). Among the latter genes were well-known JA-responsive genes, such as the gene encoding threonine deaminase 2 [40], ACX1a [41] and MYB21 [15], all being exclusively regulated by JA. The maximum of transcript accumulation correlates with the JA/JA-Ile content in wild type and *Nr* stamens, being the highest in stage 3 (Figure 3, [16]). Complementation towards wild-type transcript levels in *jai1-1 Nr* stamens of some of the in *jai1-1* deregulated genes could only be observed by trend for four genes encoding ETR6, AOX1b, the RIN-MADS box transcription factor and Cel1 in flower stage 3 and 4, respectively. These genes encode proteins, which belong to ET signaling (transcription factor such as RIN-MADS box and receptor such as ETR6), ET response (AOX1b and Cel1), and exhibited higher levels in *jai1-1* stamen of later developmental stages (stage 5 and 6) in comparison to wild type [16]. 

These rather minor effects of ET insensitivity on the gene expression of the selected genes in stamen from the double mutant did not reflect the phenotypic rescue of the mutant. This might be caused by the expression pattern of important regulatory genes exhibiting specific temporal peaks, which were not captured here. This might also explain, why a significant differentially expression between wild type and *jai1-1* could not be confirmed for all genes investigated. Alternatively, the selected genes might be not involved in regulation of the JA-mediated ET function in pollen release and stamen water content in late flower stages. Complementation of *jai1-1* defects by *Nr*-mutation might be exclusively connected to water transport and/or within the stamen tissue, since ET regulates anther dehiscence, which is predominantly related to changes in the structure and water status of the anther to facilitate complete anther opening and pollen release [17]. 

## 3. Materials and Methods 

### 3.1. Plant Material, Growth Conditions and Harvest of Stamen

*Solanum lycopersicum* plants cv. MicroTom wild type, the mutants *jai1-1* and *Nr* as well as the double mutant *jai1-1 Nr* [16] were grown in a controlled growth chamber with 16 h light (300 µmol photons·m^−2^·s^−1^) and 8 h dark, both at 28 °C and 70% humidity. Homozygous *jai1-1* plants were selected by PCR according to [14] and by checking of phenotypic markers, such as missing anthocyanin production in first leaves, protrusion of stigma. In addition, homozygous *jai1-1 Nr* were selected by germination on 200 µM ACC to select ET-insensitive plants, which do not show the typical triple response. 

Harvest of stamens was performed using 5–6 week-old plants showing the first open flowers. Stamens of developmental stages 3–6 as defined by [16] were harvested in a very strict time window of 30 min starting at 7 h after the onset of light period. Harvesting time for stamen subjected to phytohormone quantification was expanded to 90 min and stamens of primary and secondary inflorescences were used. All stamens used for hormone quantification and RNA isolation were collected on dry ice, transferred to liquid nitrogen and stored at −80 °C until further use.

### 3.2. Determination of Water Content of Stamen and Pollen Vitality

Stamen were harvested and fresh weight (FW) was determined immediately. After drying at 50 °C for one week, the dry weight (DW) was determined to calculate the water content (WC) according to the formula: WC = (FW − DW)/FW.

To determine pollen vitality, flowers of stage 6 were harvested and petals and sepals removed. Anthers were carefully opened at their tip and released pollen collected in 150 µL of MS-medium supplied with 10% (*w*/*v*) sucrose. Pollen were stained with fluorescein diacetate (Sigma-Aldrich, Munich, Germany) according to [42], simultaneously counterstained with propidium iodide (Carl Roth GmbH, Karlsruhe, Germany) and analyzed using an epifluorescence microscope AxioImager (Zeiss GmbH, Jena, Germany) equipped with the proper filter combination. Rate of vital pollen was determined from at least 900 pollen per biological replicate by counting living pollen showing strong green fluorescence upon excitation with 490 nm and dead pollen showing red fluorescence due to fluorescein and propidium iodide accumulation, respectively (see Figure S1). 

### 3.3. Determination of ACC, JA and JA-Ile

The quantification of ACC, JA and JA-Ile was carried out with homogenized stamen samples. To determine content of ACC, 500 mg of material was extracted with 10 mL methanol supplied with 50 ng [^2^H_4_]-ACC as internal standard according to [16]. Briefly, the homogenate was purified by two subsequent purification steps using a column filled with DEAE-Sephadex A25 (GE Healthcare, Solingen, Germany) and a LiChrolutRP-18-column (Merck, Darmstadt, Germany). The resulting eluate was dissolved in chloroform:N,N-diisopropylethylamine (1:1, *v*/*v*) and derivatized with pentafluorobenzylbromide at 20 °C overnight. After evaporation, samples were dissolved in 5 mL n-hexane and passed through a Chromabond-SiOH column (Machery-Nagel, Düren, Germany). The pentafluorobenzyl esters were eluted with n-hexane:diethylether (2:1, *v*/*v*), evaporated, dissolved in acetonitrile and analyzed by GC-MS as described by [43].

For quantification of jasmonates, the extraction was done using approximately 50 mg of material and 500 μL methanol supplied with 50 ng [^2^H_6_] JA and 50 ng [^2^H_2_] JA-Ile as internal standards. The solid phase extraction was carried out on a 96 HR XC well plate (Macherey & Nagel) with the final elution by 900 μL acetonitrile. The phytohormone measurement was carried out with 10 μL of each eluate by UPLC-MS/MS according to [44]. 

The contents of ACC, JA, and JA-Ile were calculated using the ratio of analyte and internal standard peak heights from at least four biological replicates each.

### 3.4. Extraction of Metabolites and Separation by High Performance Thin-Layer-Chromatography (HPTLC) and Liquid Chromatography-Mass Spectroscopy (LC-MS)

Stamens of the developmental stage 6 were collected and 60 mg material directly homogenized in 200 µL 90% (*v*/*v*) methanol and incubated in a sonication bath (Transsonic 460, Elma, Singen, Germany) at 35 kHz for 15 min. Samples were centrifuged at 20,817× *g* and 4 °C for 15 min and the supernatants were diluted in a ratio of 1:3 or 1:10 with 90% (*v*/*v*) methanol. 

For HPTLC analysis, 20 µL of the 1:3 diluted methanolic stamen extracts were loaded on a 20 cm × 10 cm pre-coated silica NanoAdamant UV_254_ thin-layer chromatography plate (Macherey-Nagel). For comparison, 10 µL of each standard (0.5 mM rutin, 0.5 mM feruloylquinic acid, 0.5 mM chlorogenic acid) were also spotted on the TLC-plate. As a mobile phase, a mixture of ethylacetate:formic acid:acetic acid:water (100:11:11:27) was used. Plates were stained with 1% (*w*/*v*) 2-aminoethyl diphenylborate (DPBA) and analyzed under UV light at 366 nm before and after derivatization with DPBA.

LC-MS analysis was done with the 1:10 diluted methanolic extract. 10 µL of the extracts were separated on a 5 cm RP18 Nucleoshell column (Marchery-Nagel) using a HPLC system from Waters including the Waters e2695 separation module (Waters, Eschborn, Germany) at a flow rate of 0.6 mL min^−1^ with a gradient from 10% (*v*/*v*) B in A to 45% (*v*/*v*) B in A within 9 min. As solvents 0.1% formic acid (A) and acetonitrile (B) were used. UV-absorbance was detected by the Waters 2998 PDA detector at 354 nm. Eluted compounds were monitored by the Acquity QDA Detector System from Waters in a range from *m*/*z* 150 to 800 Da using the Negative Scan Mod and 15 V Cone Voltage. 

### 3.5. Preparation of the Feruloylquinic Acid Standard

The feruloylquinic acid used as reference compound in HPTLC and LC-MS-analysis was enzymatically synthesized using the phenylpropanoid and flavonoid *O*-methyltransferase (PFOMT) from ice plant [45]. Purified recombinant protein was kindly provided by Thomas Vogt (IPB, Halle, Germany). The assay (total volume of 1 mL) consisted of 50 mM KPi buffer (pH 7.5) supplied with 10% (*v*/*v*) glycerol, 20 µM chlorogenic acid, 500 µM *S*-adenosyl-L-methionine, 200 µM ascorbic acid, 200 µM MgCl_2_, and 71.2 µg purified protein. The reaction was run at 37 °C for 10 min and stopped by adding 1 mL water. The products were purified by solid phase extraction using an amphiphillic Oasis^®^ HLB3cc column (Waters, Milford, MA, USA). All fractions were monitored by HPLC-MS. The feruloylquinic acid was eluted with 80% (*v*/*v*) methanol and concentrated by rotary evaporation. The product was an isomeric product mixture of feruloylquinic acid, since PFOMT has the catalytic activity to methylate chlorogenic acid at positions 3 and 4. 

### 3.6. Isolation of RNA and RT-qPCR based on Nanofluidic Arrays

RNA isolation from homogenized stamen material was performed using the RNeasy Plant Mini Kit (Qiagen, Hilden, Germany) according to the supplier’s instructions followed by DNase treatment using DNA-free Kit (Thermo Fisher Scientific, Waltham, MA, USA). RNA quality was tested by capillary electrophoresis using QIAxcel Advanced System (Qiagen). All RNA samples were diluted to 90 ng RNA per µL.

Determination of transcript accumulation was done using the BioMark™ system with a “48.48 Dynamic Array Chip for Gene Expression” (Fluidigm, San Francisco, CA, USA) according to the supplier’s instructions. First strand cDNA synthesis was performed using 5× Reverse Transcription Master Mix (Fluidigm) and was followed by pre-amplification of cDNA. For this, 2 µL of all gene-specific primer pairs (50 µM each primer, see Supplemental Table S2) were added to 104 µL DNA suspension buffer (Fluidigm) to get a final concentration of 500 nM for all primers. Pre-amplification was done in a Thermocycler (Bio-Rad, Munich, Germany) using a touch-down PCR with steps of 1 °C from 65 °C down to 60 °C followed by nine cycles at 60 °C. To remove remaining primers, the reaction mix was treated with exonuclease I (New England Biolabs, Frankfurt/M., Germany) according to the protocol from Fluidigm. After a 1:5 dilution with TE buffer, the amplified cDNA was mixed with 2× SsoFast EvaGreen Supermix with Low ROX (Bio-Rad) and 20× DNA Binding Dye Sample Loading Reagent (Fluidigm) to get the ‘Sample Mix’. Mixtures consisting of 22.5 µL 2× Assay Loading Reagent, 22.5 µL 1× DNA Suspension Buffer and 5 µL of a 50 µM solution of the respective forward and reverse primers served as ‘Assay Mix’. Five µL of each ‘Sample Mix’ and each ‘Assay mix’ were loaded onto the chip, which has been primed before according to the supplier’s instructions. PCR was run in the BioMark™ system (Fluidigm) using the following temperature regime: 50 °C for 2 min, 95 °C for 10 min, and 35 cycles of 95 °C for 15 s—56 °C for 30 s—72 °C for 30 s. The reaction was finished by obtaining melting curves by subjecting it to 60 °C for 30 sec followed by a rise in temperature from 60 to 95 °C with steps of 1 °C per s. 

Data quality was checked using the software from the BioMark™ system. Data not fulfilling the respective quality criteria or delivering numbers below the “quality threshold” of 0.65 were not considered for calculating the relative transcript levels. Relative transcript levels were calculated by the comparative Cq method [46] using *SlTIP41* [47] as the constitutively expressed gene. For all four developmental stages from all four genotypes, three independent biological replicates were used resulting in 48 samples.

### 3.7. Statistical Analysis

All data were analyzed by one- factorial analysis of variance (ANOVA). As multiple comparison test served Tukey’s HSD post hoc test. Differences were considered significant at a probability level of *p* ≤ 0.05. All statistical analyses were performed using the R software (R Core Team; https://www.r-project.org/).

## 4. Conclusions

Next to their role in development of female organs of tomato [14,15], jasmonates are also important regulators of stamen and pollen development, thereby contributing to pollen nutrition at early developmental stages and regulating the biosynthesis and action of ET at late developmental stages [16]. The data obtained from stamen of the double mutant *jai1-1 Nr* showing insensitivity to JA and ET supported the conclusion that at early stages, which are not related to ET function, *jai1-1* dominates *Nr* and introduction of ET insensitivity did not change the *jai1-1* phenotype. This is in contrast to late developmental stages, in which ET insensitivity resulted in a rescue of water content (dehiscence) and a partial rescue in pollen vitality and accumulation of phenylpropanoids. These data confirm the role of jasmonates in controlling ET function in late stamen development, which is mainly related to anther dehiscence and pollen release, but also affects pollen vitality to some extent. This is in strong contrast to Arabidopsis, where JA insensitivity and deficiency result in nonvital pollen and delayed anther dehiscence [48], and high JA concentrations induce precocious anther dehiscence [49]. 

## Supplementary Materials

The following are available online at https://www.mdpi.com/2223-7747/8/8/277/s1, Figure S1: Pollen stained with fluorescein-diacetate and propidium iodide to determine their vitality. Figure S2: LC-MS chromatograms of authentic standards. Table S1: Relative transcript levels of JA and ET-regulated genes in four developmental stages of stamens from wild type, *Nr*, *jai1-1*, and *jai1-1 Nr*. Table S2: Primer sequences used for RT-qPCR.
