# Supplementary material for: Effects of Jasmonate on Ethylene Function during the Development of Tomato Stamens
Source: Plants (Basel). 2019 Aug 9;8(8):277. doi: 10.3390/plants8080277 (PMC6724093; doi:10.3390/plants8080277)
Supplement: Supplementary file 1 [file plants-08-00277-s001.pdf]

Supplemental materials

Supplemental Figures:

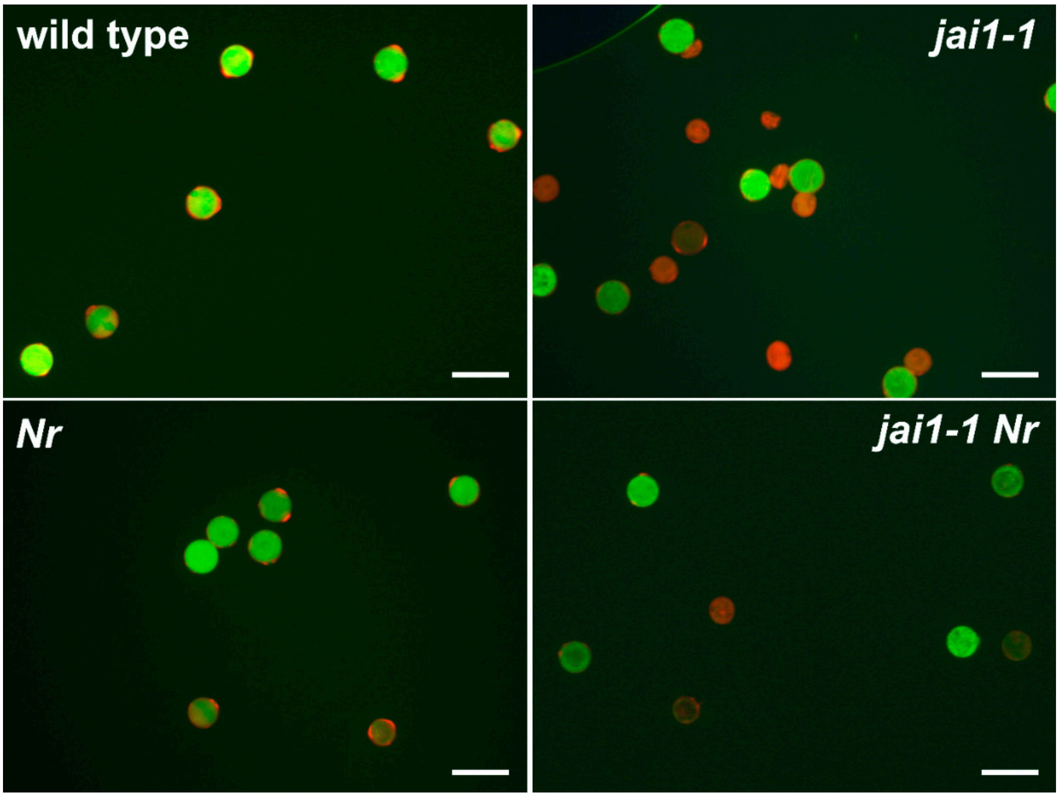

Figure S1: Pollen stained with fluorescein-diacetate and propidium iodide to determine their vitality.

Representative micrographs of pollen isolated from open flowers of the respective genotypes. Note that the majority of pollen from wild type and *Nr* exhibit green fluorescence, which is indicative for living cells, whereas a reasonable number of pollen from *jai1-1* and *jai1-1 Nr* shows red fluorescence due to the staining with propidium iodide indicative for dead cells. Bars represent 20 µm.

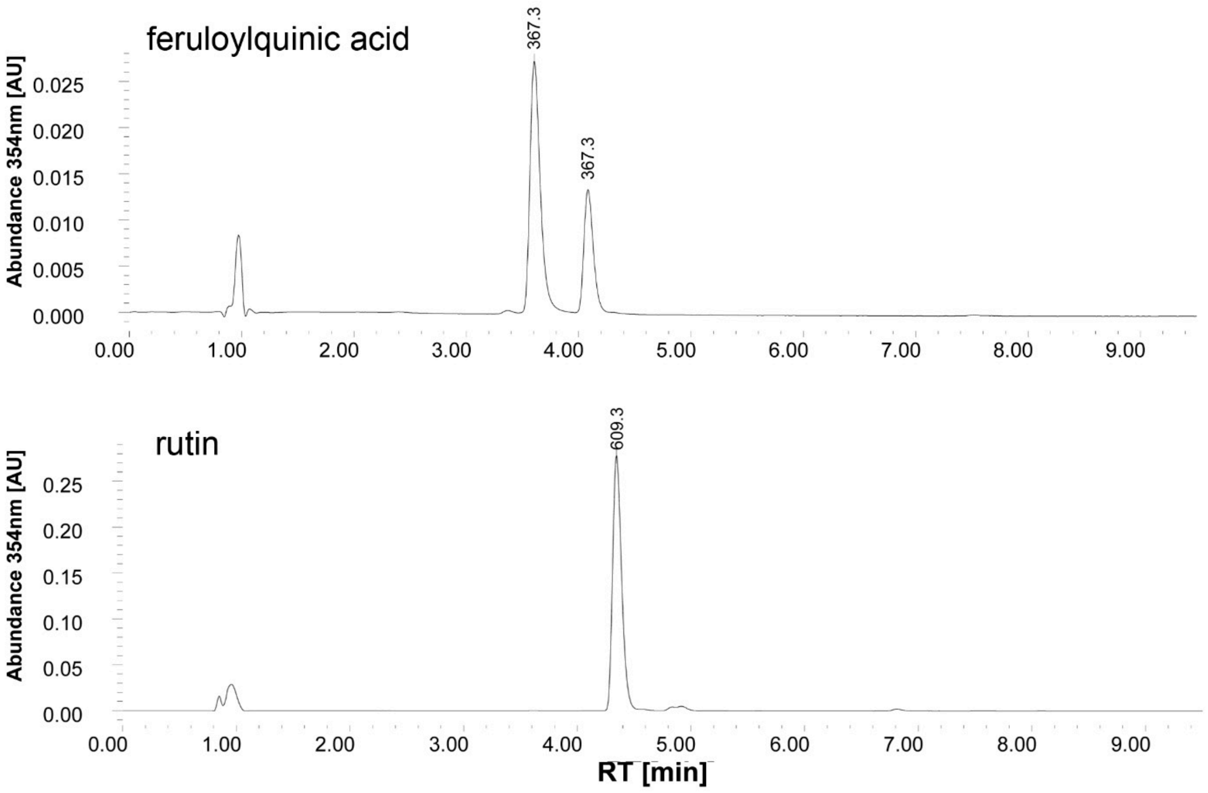

Figure S2: LC-MS chromatograms of authentic standards.

Feruloylquinic acid was produced enzymatically using the phenylpropanoid and flavonoid O-methyltransferase (PFOMT) from ice plant Vogt et al. (2004) and resulted in an isomeric product mixture visible by two peaks with identical m/z ratio.

Supplemental Tables:

Table S1: Relative transcript levels of JA and ET-regulated genes in four developmental stages of stamens from wild type, *Nr*, *jai1-1*, and *jai1-1 Nr*.
All transcript levels were determined by RT-qPCR using Benchmark system (Fluidigm) and set in relation to SlTIP41. Mean values ± SE are presented. Different letters designate statistically different values (1-factorial ANOVA followed by Tukey's HSD test; p ≤ 0.05).

stage 3	**wild type**		***Nr***		***jai1-1***		***jai1-1 Nr***	
**ACC-Ox**	0.4712 ± 0.2669	a	0.3928 ± 0.0597	a	2.8207 ± 0.6424	a	2.9967 ± 0.2986	a
**ACC-Ox5**	0.2524 ± 0.0391	a	0.2636 ± 0.1308	a	1.5049 ± 0.2921	a	1.8506 ± 0.3273	a
**ACX1**	8.7923 ± 1.3716	a	7.9017 ± 1.0758	a	5.7808 ± 0.5301	a	3.9620 ± 0.7384	a
**AOX1b**	0.0020 ± 0.0008	a	0.0021 ± 0.0012	a	0.0777 ± 0.0122	a	0.0293 ± 0.0052	a
**bXylo**	0.5002 ± 0.1589	a	0.7065 ± 0.3863	a	1.4225 ± 0.5296	a	2.5241 ± 0.3546	a
**AP2/ERF**	0.0143 ± 0.0015	a	0.0369 ± 0.0332	a	0.0976 ± 0.0797	a	0.1815 ± 0.1089	a
**ETR6**	**0.0100 ± 0.0035**	**a**	**0.0019 ± 0.0010**	**a**	**0.3510 ± 0.0785**	**b**	**0.1027 ± 0.0315**	**b**
**GRAS**	0.0256 ± 0.0250	a	0.0018 ± 0.0018	a	0.0002 ± 0.0002	a	0.0002 ± 0.0002	a
**IAA-aaHy**	**4.9023 ± 0.6273**	**a**	**0.8181 ± 0.8181**	**a**	**0.0701 ± 0.0446**	**b**	**0.0657 ± 0.0258**	**b**
**JAZ2**	1.6955 ± 0.4822	a	0.6412 ± 0.6412	a	0.2809 ± 0.0858	a	0.1532 ± 0.1122	a
**JAZ9**	0.0280 ± 0.0155	a	0.0090 ± 0.0056	a	0.0000	a	0.0000	a
**LAP**	18.2448 ± 16.2373	a	6.2946 ± 4.7610	a	0.0002 ± 0.0001	a	0.0002 ± 0.0002	a
**Cel1**	0.2951 ± 0.1327	a	0.4673 ± 0.1104	a	14.2057 ± 7.3152	a	9.6162 ± 4.3681	a
**MADS**	0.5266 ± 0.0959	a	0.2604 ± 0.0464	a	0.6050 ± 0.1024	a	0.6002 ± 0.0547	a
**Meta7**	0.0416 ± 0.0203	a	0.0277 ± 0.0047	a	0.3369 ± 0.1545	a	0.2582 ± 0.2157	a
**MPF2**	0.0005 ± 0.0003	a	0.0002 ± 0.0002	a	0.1130 ± 0.0086	a	0.0393 ± 0.0140	a
**MYB21**	29.1981 ± 5.8365	a	15.3240 ± 8.0706	a	7.3673 ± 0.7459	a	4.9896 ± 0.5975	a
**MYBAN2**	0.0088 ± 0.0088	a	0.0000	a	0.0003 ± 0.0002	a	0.0000	a
**MYB-like**	0.0218 ± 0.0081	a	0.0233 ± 0.0112	a	0.0119 ± 0.0024	a	0.0120 ± 0.0002	a
**Pirin**	0.0481 ± 0.0204	a	0.0238 ± 0.0075	a	0.2439 ± 0.0386	a	0.2663 ± 0.0680	a
**RIN**	0.0016 ± 0.0015	a	0.0003 ± 0.0003	a	0.0942 ± 0.0433	a	0.0170 ± 0.0131	a
**Subti**	0.0015 ± 0.0014	a	0.0122 ± 0.0103	a	0.0039 ± 0.0015	a	0.0115 ± 0.0075	a
**TD2**	**106.2915 ± 54.4440**	**a**	**63.3677 ± 41.5357**	**a**	**0.2753 ± 0.0236**	**b**	**0.2242 ± 0.0430**	**b**
**TrypSyn**	**1.0661 ± 0.3713**	**a**	**0.5042 ± 0.1466**	**a**	**0.0013 ± 0.0006**	**b**	**0.0043 ± 0.0022**	**b**
**Xyloglu3**	0.0680 ± 0.0078	a	0.8330 ± 0.7316	a	0.3816 ± 0.2328	a	0.2128 ± 0.0972	a

stage 4	**wild type**		***Nr***		***jai1-1***		***jai1-1 Nr***	
**ACC-Ox**	**0.5790 ± 0.1487**	**a**	**0.3261 ± 0.0249**	**a**	**1.7501 ± 0.2114**	**b**	**2.5540 ± 0.2974**	**b**
**ACC-Ox5**	1.0232 ± 0.4579	a	0.0876 ± 0.0219	a	0.3344 ± 0.1542	a	0.2311 ± 0.0560	a
**ACX1**	12.5988 ± 1.2091	a	8.6602 ± 1.0442	a	3.3553 ± 0.5349	a	3.8885 ± 0.9098	a
**AOX1b**	0.1264 ± 0.0507	a	0.0148 ± 0.0105	a	0.0164 ± 0.0086	a	0.0195 ± 0.0043	a
**bXylo**	0.1852 ± 0.0281	a	0.0884 ± 0.0240	a	0.1874 ± 0.0588	a	0.3600 ± 0.0235	a
**AP2/ERF**	**0.0562 ± 0.0562**	**ab**	**0.0505 ± 0.0151**	**a**	**0.7321 ± 0.0970**	**b**	**0.6936 ± 0.0657**	**b**
**ETR6**	0.2600 ± 0.0987	a	0.0138 ± 0.0066	a	0.1020 ± 0.0442	a	0.0185 ± 0.0021	a
**GRAS**	0.0002 ± 0.0001	a	0.0000	a	0.0000 ± 0.0000	a	0.0000	a
**IAA-aaHy**	**2.0758 ± 1.4523**	**a**	**4.1320 ± 0.1704**	**a**	**0.0188 ± 0.0014**	**b**	**0.0505 ± 0.0080**	**b**
**JAZ2**	6.2075 ± 2.1313	a	1.9820 ± 0.6015	a	0.3705 ± 0.1855	a	0.9623 ± 0.2046	a
**JAZ9**	0.0036 ± 0.0021	a	0.0135 ± 0.0047	a	0.0000	a	0.0001 ± 0.0001	a

**LAP**	0.5813 ± 0.0247	a	0.3575 ± 0.1003	a	0.0000	a	0.0004 ± 0.0004	a
**Cel1**	**5.7649 ± 2.5183**	**a**	**0.5008 ± 0.1101**	**b**	**43.7659 ± 9.3487**	**c**	**20.2118 ± 3.5641**	**c**
**MADS**	1.0425 ± 0.1032	a	0.3703 ± 0.0325	a	0.7739 ± 0.1311	a	0.7415 ± 0.1153	a
**Meta7**	0.3845 ± 0.2224	a	0.0216 ± 0.0015	a	0.2142 ± 0.0515	a	0.4280 ± 0.1747	a
**MPF2**	0.0001 ± 0.0001	a	0.0005 ± 0.0002	a	0.0628 ± 0.0158	a	0.0247 ± 0.0046	a
**MYB21**	**42.7316 ± 5.0046**	**a**	**36.1562 ± 1.4711**	**a**	**9.6939 ± 2.0089**	**b**	**8.7854 ± 0.1753**	**b**
**MYBAN2**	0.0000	a	0.0000	a	0.0002 ± 0.0001	a	0.0000	a
**MYB-like**	0.0160 ± 0.0087	a	0.0051 ± 0.0004	a	0.0114 ± 0.0022	a	0.0130 ± 0.0018	a
**Pirin**	0.0859 ± 0.0339	a	0.0167 ± 0.0040	a	0.1004 ± 0.0404	a	0.0514 ± 0.0122	a
**RIN**	0.0000	a	0.0001 ± 0.0001	a	0.0365 ± 0.0144	a	0.0660 ± 0.0226	a
**Subti**	0.0012 ± 0.0010	a	0.0014 ± 0.0004	a	0.0029 ± 0.0009	a	0.0045 ± 0.0024	a
**TD2**	**66.6793 ± 13.9023**	**a**	**59.1867 ± 13.5608**	**a**	**0.1075 ± 0.0146**	**b**	**0.0660 ± 0.0038**	**b**
**TrypSyn**	0.5211 ± 0.4685	a	0.6681 ± 0.1252	a	0.0006 ± 0.0006	a	0.0008 ± 0.0007	a
**Xyloglu3**	0.2386 ± 0.0241	a	0.1118 ± 0.0562	a	0.8262 ± 0.3004	a	0.7999 ± 0.0415	a

stage 5	**wild type**		***Nr***		***jai1-1***		***jai1-1 Nr***	
**ACC-Ox**	1.9981 ± 0.5095	a	1.6077 ± 0.3645	a	1.1837 ± 0.0855	a	1.5942 ± 0.1095	a
**ACC-Ox5**	0.5168 ± 0.2451	a	0.0450 ± 0.0232	a	0.0264 ± 0.0139	a	0.0300 ± 0.0067	a
**ACX1**	**14.4046 ± 3.2464**	**a**	**8.3851 ± 1.0674**	**a**	**3.8525 ± 0.8319**	**b**	**3.1069 ± 0.1571**	**b**
**AOX1b**	0.3221 ± 0.0222	a	0.0888 ± 0.0123	a	0.0166 ± 0.0066	a	0.0032 ± 0.0017	a
**bXylo**	0.1503 ± 0.0449	a	0.4521 ± 0.0742	a	0.0580 ± 0.0437	a	0.0627 ± 0.0114	a
**AP2/ERF**	0.6109 ± 0.3968	a	0.4740 ± 0.2376	a	0.8824 ± 0.0897	a	0.8802 ± 0.0247	a
**ETR6**	0.0503 ± 0.0271	a	0.0096 ± 0.0040	a	0.0678 ± 0.0231	a	0.0054 ± 0.0014	a
**GRAS**	0.0003 ± 0.0003	a	0.0001 ± 0.0001	a	0.0002 ± 0.0002	a	0.0000	a
**IAA-aaHy**	0.0535 ± 0.0304	a	0.0088 ± 0.0031	a	0.0131 ± 0.0062	a	0.0081 ± 0.0063	a
**JAZ2**	9.2644 ± 1.9052	a	28.4300 ± 23.5365	a	0.5977 ± 0.3013	a	0.8518 ± 0.0587	a
**JAZ9**	0.0003 ± 0.0003	a	0.0000	a	0.0000	a	0.0000	a
**LAP**	0.2704 ± 0.2193	a	0.0280 ± 0.0105	a	0.0008 ± 0.0004	a	0.0000	a
**Cel1**	15.8777 ± 8.1324	a	12.4718 ± 3.4393	a	28.8038 ± 5.4346	a	27.6376 ± 4.5335	a
**MADS**	0.9732 ± 0.2834	a	0.6556 ± 0.0377	a	0.9217 ± 0.1852	a	0.7008 ± 0.0442	a
**Meta7**	1.1400 ± 0.5761	a	0.3267 ± 0.0530	a	0.0761 ± 0.0300	a	0.0718 ± 0.0071	a
**MPF2**	0.0189 ± 0.0185	a	0.0003 ± 0.0003	a	0.0304 ± 0.0014	a	0.0140 ± 0.0028	a
**MYB21**	**42.3775 ± 11.1604**	**a**	**51.7808 ± 3.8583**	**a**	**14.4465 ± 1.6961**	**b**	**12.8757 ± 0.4711**	**b**
**MYBAN2**	0.0000	a	0.0002 ± 0.0002	a	0.0002 ± 0.0001	a	0.0000	a
**MYB-like**	0.0183 ± 0.0060	a	0.0122 ± 0.0034	a	0.0064 ± 0.0028	a	0.0119 ± 0.0008	a
**Pirin**	0.1416 ± 0.0077	a	0.0260 ± 0.0055	a	0.0505 ± 0.0142	a	0.0366 ± 0.0034	a
**RIN**	**0.0000**	**a**	**0.0000**	**a**	**0.0246 ± 0.0095**	**b**	**0.0070 ± 0.0033**	**b**
**Subti**	0.0003 ± 0.0002	a	0.0007 ± 0.0004	a	0.0005 ± 0.0005	a	0.0005 ± 0.0005	a
**TD2**	**24.2551 ± 6.9759**	**a**	**27.0128 ± 2.6992**	**a**	**0.2575 ± 0.0595**	**b**	**0.1458 ± 0.0090**	**b**
**TrypSyn**	0.0005 ± 0.0002	a	0.0001 ± 0.0000	a	0.0001 ± 0.0001	a	0.0000 ± 0.0000	a
**Xyloglu3**	0.5471 ± 0.1633	a	0.1062 ± 0.0256	a	0.1055 ± 0.0455	a	0.0914 ± 0.0152	a

stage 6	**wild type**		***Nr***		***jai1-1***		***jai1-1 Nr***	
**ACC-Ox**	7.2079 ± 1.0903	a	3.0021 ± 0.5001	a	4.1536 ± 2.2147	a	4.2906 ± 1.9331	a
**ACC-Ox5**	0.0174 ± 0.0053	a	0.0256 ± 0.0047	a	0.0268 ± 0.0145	a	0.0392 ± 0.0052	a

**ACX1**	7.3001 ± 1.0809	a	5.7879 ± 1.5728	a	3.9358 ± 0.3620	a	4.7079 ± 0.6227	a
**AOX1b**	0.0375 ± 0.0101	a	0.0317 ± 0.0171	a	0.0531 ± 0.0217	a	0.0236 ± 0.0147	a
**bXylo**	0.0878 ± 0.0151	a	0.3219 ± 0.1073	a	0.0351 ± 0.0147	a	0.0138 ± 0.0057	a
**AP2/ERF**	0.2648 ± 0.1399	a	0.6146 ± 0.0656	a	3.2172 ± 1.7817	a	1.2984 ± 0.2549	a
**ETR6**	0.0213 ± 0.0096	a	0.0023 ± 0.0005	a	0.9121 ± 0.7228	a	0.0167 ± 0.0095	a
**GRAS**	**0.0005 ± 0.0002**	**a**	**0.0000**	**b**	**0.0000**	**b**	**0.0000**	**b**
**IAA-aaHy**	0.0203 ± 0.0053	a	0.0048 ± 0.0008	a	0.0071 ± 0.0051	a	0.0022 ± 0.0017	a
**JAZ2**	11.6272 ± 2.0629	a	7.1288 ± 3.5678	a	1.1900 ± 0.6067	a	2.9077 ± 0.3890	a
**JAZ9**	0.0002 ± 0.0002	a	0.0000	a	0.0000	a	0.0000	a
**LAP**	0.0397 ± 0.0167	a	0.0215 ± 0.0124	a	0.0044 ± 0.0030	a	0.0003 ± 0.0003	a
**Cel1**	1.2831 ± 0.4853	a	4.1201 ± 0.4031	a	15.9382 ± 8.2183	a	7.8820 ± 4.0619	a
**MADS**	1.3139 ± 0.0312	a	0.6669 ± 0.0496	a	1.5920 ± 0.1956	a	0.8829 ± 0.1048	a
**Meta7**	0.0269 ± 0.0133	a	0.1056 ± 0.0450	a	0.0052 ± 0.0013	a	0.0149 ± 0.0080	a
**MPF2**	0.0008 ± 0.0004	a	0.0005 ± 0.0005	a	0.0120 ± 0.0060	a	0.0124 ± 0.0025	a
**MYB21**	23.8062 ± 2.5866	a	27.2789 ± 3.8392	a	17.2325 ± 1.6721	a	9.5256 ± 4.7683	a
**MYBAN2**	0.0012 ± 0.0005	a	0.0000 ± 0.0000	a	0.0509 ± 0.0255	a	0.0018 ± 0.0016	a
**MYB-like**	0.0603 ± 0.0092	a	0.0181 ± 0.0014	a	0.0069 ± 0.0028	a	0.0095 ± 0.0032	a
**Pirin**	0.0246 ± 0.0090	a	0.0102 ± 0.0026	a	0.3517 ± 0.2101	a	0.2357 ± 0.0803	a
**RIN**	0.0000	a	0.0000	a	0.0770 ± 0.0326	a	0.0564 ± 0.0181	a
**Subti**	0.0015 ± 0.0007	a	0.0009 ± 0.0004	a	0.0040 ± 0.0020	a	0.0005 ± 0.0003	a
**TD2**	11.5470 ± 2.3231	a	15.5387 ± 2.2769	a	6.8829 ± 5.9233	a	0.7639 ± 0.2330	a
**TrypSyn**	0.0023 ± 0.0016	a	0.0001 ± 0.0000	a	0.0000 ± 0.0000	a	0.0004 ± 0.0003	a
**Xyloglu3**	0.1523 ± 0.0407	a	0.1477 ± 0.0376	a	0.4362 ± 0.3254	a	0.1225 ± 0.0454	a

Table S2: Primer sequences for Fluidigm-qRT-PCR analysis with JA-regulated genes in stamen.

**Gene (Abbreviation)**	**Sequence [5'->3']**	
TIP41 (reference gene)	*for_*	TCA GTG GGA GGA TTG TAA GG
(Solyc10g049850)	*rev_*	GGT TCT TTA GAC GCC AAT GC
JAZ2	*for_*	CTG ATC AAT CTG GTG TGA GTT TTG
(Solyc12g009220)	*rev_*	CAG AAG GCT GTG GCA TTG AC
JAZ9	*for_*	CAA GTA GAG GAA TGG AGA TG
(Solyc08g036640)	*rev_*	ATG GTG ATG AAG GCT CAG AC
Leucin aminopeptidase (***LAP***)	*for_*	CCT GGT AAT GGC GGT GCT ATA A
(Solyc12g010040)	*rev_*	TCG AGA TGC AAC CAC TGA ACC
Threonin deaminase 2 (***TD2***)	*for_*	CTT TAT GCC GTT ACC GTA ATC AGG
(Solyc09g008670)	*rev_*	GGA ATC TGG AAT CCC ATC AAC A
MYB21	*for_*	TGC TGG TCT CAA ACG TAC TG
(Solyc02g067760)	*rev_*	TAG CCA TCG GAG TCT ACA AC
ACX1A	*for_*	CAA ATG CTG TAT CAC TGG TTG ATG
(Solyc08g078390)	*rev_*	CCA TCA TAA CGT CCA AGA ATT GAA C
Pirin	*for_*	CCT GGA ATG CCT TCC TCT AC
(Solyc09g098160)	*rev_*	AGA ACC AAA CGC TCC CTC TC
AOX1b	*for_*	CGG GAC CTA AAT CAC TTT GC
(Solyc08g075550)	*rev_*	AAC TCA TGC CCT TGA CAC TG
Metacaspase 7 (Meta7)	*for_*	AGC CAC TTA AAC AAG ATG AG
(Solyc10g081300)	*rev_*	TCT TCA TTA GCT TGA CAT CC
Subtilisin-like Protease (**Subti**)	*for_*	TGA TGG TCT GCC AGA AAT CC
(Solyc01g087800)	*rev_*	GGG CCT ACA AAT TCC TTT CC
Xyloglucan endotransglucosylase-hydrolase 3 (**Xyloglu3**)	*for_*	CCA AAG AAC CAA CCC ATG AG
(Solyc03g093130)	*rev_*	AGC CCA ATC ATC AGC ATT CC
Endo-1,4-β-glucanase (***Cel1***)	*for_*	GGA TGG CCC ATG GCT TAT TC
(*Solyc08g081620*)	*rev_*	CAA TAG CAG CCC AAC TCA AC
MADS-Box MPF2-like TF (**MPF2**)	*for_*	GCC TTT AGT GAC TGA CAT GG
(*Solyc04g076280*)	*rev_*	TAG ACT CAG ATG ATT GGC CC
MADS-Box TF (**MADS**)	*for_*	GAA CTT GGA GAA CCA ACT GG
(*Solyc04g076700*)	*rev_*	TTC TTA CAC GAA CAC CAC GG
ACC-Oxidase 5 (**ACC-Ox5**)	*for_*	GGA CAA TGT GAA GCA GTT CG
(*Solyc07g026650*)	*rev_*	GCT TCT TCA TGT TGG CTT CG
IAA-aminoacid-hydrolase (**IAA-aaHy**)	*for_*	ATA GTT GCA TCT AGG GCT GG
(*Solyc05g006220*)	*rev_*	TGT GGT ATA GCA GCA TGT CC
Tryptophan-Synthase (**TrypSyn**)	*for_*	GAC TAG GAA ACA AGC CAT GG
(*Solyc10g005320*)	*rev_*	AAC ACA TGT GGT TTG CCA CC
ACC-Oxidase (**ACC-Ox**)	*for_*	TCA GTG GTC TCC AAC TAC TC
(*Solyc02g036350*)	*rev_*	GCA TCG GTG GAA CAT CAA TC
RIN MADS-Box TF (***RIN***)	*for_*	CAA ACA TCA TGG CAT TGT GG
(*Solyc05g012020*)	*rev_*	ATG AGA AGG CTG TTC ATG TC
ETR6	*for_*	GCT GCA GTG GTT GAA GAA TC
(*Solyc09g089610*)	*rev_*	TTC TGT TCC GTC AAC CTG TC
AP2/ERF TF	*for_*	CTT CTT TCA GCC ACA AAC TC
(*Solyc04g071770*)	*rev_*	GCT GTT GCT ACT GCT ATT GC
MYB-like TF	*for_*	GAC GAG ACT ACA CCA AAT CG
(*Solyc05g052610*)	*rev_*	CAA GAA CTG ATT TCC CAG GG
MYB-TF AN2 (**MYBAN2**)	*for_*	CAT CTT GTT CCT GCT AGA GC
(*Solyc10g086250*)	*rev_*	CTG CAA CTC TTT CGA CAT CG
GRAS-family TF (**GRAS**)	*for_*	TGC TAG TAG ATG AAG ACG CG
(*Solyc02g092570*)	*rev_*	CCG ATC TTA ATC TGC ACA CC
